# Special Issue “Molecular Advances in Obstetrical and Gynaecological Disorders”

**DOI:** 10.3390/ijms27010492

**Published:** 2026-01-03

**Authors:** Panagiotis Christopoulos, Anna Eleftheriades

**Affiliations:** 2nd Department of Obstetrics and Gynecology, Medical School, National and Kapodistrian University of Athens, 115 28 Athens, Greece

Gynecological and obstetrical conditions are among the most prevalent and significant health conditions affecting women globally. These disorders span the entire reproductive timeline and beyond, including developmental anomalies, infertility, complications during pregnancy, and malignancies. Their impact is profound: not only on women’s quality of life and expectancy, but also on the health of future generations. Despite major advances in diagnostic and therapeutic methods, the mechanisms of many of these disorders remain poorly understood, limiting our ability to implement early interventions and personalized care.

Recent decades have witnessed remarkable advances in molecular biology that have begun to transform the field of obstetrics and gynecology. Genomics, transcriptomics, proteomics, metabolomics, and epigenetics are now central to understanding the complex pathophysiology of these disorders. Such molecular approaches are leading to more accurate disease classification, earlier diagnosis, targeted therapies, and better prognostic indicators.

This Special Issue, “Molecular Advances in Obstetrical and Gynecological Disorders”, aims to showcase both original research and comprehensive reviews that illustrate the potential of molecular research in women’s health. Our aim is to bridge the gap between basic science and clinical practice, highlighting how molecular-level discoveries can inform more effective diagnosis, treatment, and prevention strategies.

One major focus of the Special Issue is molecular diagnostics, which continues to evolve rapidly. The latest research explores how molecular biomarkers can aid in the early detection and monitoring of gynecological diseases. For example, cervical cancer, despite being highly preventable, remains a leading cause of mortality among women. Advances in molecular cytology and research on molecular markers associated with human papillomavirus (HPV) infection and disease progression have led to better understanding of the pathophysiology of cervical metaplasia, dysplasia and cancer. Innovations in molecular cytology and the discovery of distinct gene expression patterns are helping to refine screening methods and paving the way for more sensitive and specific screening strategies [1].

The role of microRNAs (miRNAs) and other non-coding RNAs in reproductive pathology constitutes another key area highlighted in this issue. These small regulatory molecules have emerged as promising candidates for non-invasive diagnostics, especially in diseases like endometriosis. By profiling circulating miRNAs, researchers are now uncovering disease-specific molecular signatures that could potentially reduce diagnostic delays and improve patient outcomes [2,3].

The Special Issue also highlights the genetic and epigenetic foundations of uncommon developmental conditions, including Mayer–Rokitansky–Küster–Hauser (MRKH) Syndrome. This congenital condition, characterized by the underdevelopment or absence of the uterus and upper vaginal tract has been linked to a combination of single-gene mutations and epigenetic changes that disrupt the development of the Müllerian ducts during gestation. New research sheds light on potential causative gene pathways and epigenetic alterations, offering hope for better understanding, and ultimately, more effective management [4].

In the domain of reproductive endocrinology, molecular biology is offering new perspectives on prevalent disorders like polycystic ovary syndrome (PCOS), anovulation, and premature ovarian insufficiency (POI). These conditions are often linked to metabolic dysregulation and hormonal imbalance, but their underlying causes are heterogeneous and complex. The molecular mechanisms behind PCOS, anovulation, and POI involve a complex interplay of genetics, epigenetics, hormonal signaling, and environmental factors. PCOS is linked to genetic variants in specific genes, androgen excess and impaired insulin signaling all leading to follicular dysfunction and anovulation. Anovulation is the central feature, resulting from these disruptions and leading to chronic infertility. POI is often caused by genetic defects in genes such as FOXL2, BMP15, and FMR1, epigenetic changes, DNA damage repair issues, or autoimmune causes, leading to premature loss of ovarian function and follicles [5,6]. Through advanced genomic and transcriptomic studies, researchers are beginning to differentiate distinct molecular subtypes of PCOS, which may help tailor interventions based on individual molecular profiles, and similarly, studies on the genetic regulation of ovarian function are contributing to the development of targeted fertility preservation strategies [7].

Pregnancy-related disorders such as gestational diabetes, preeclampsia (PE), and fetal growth restriction (FGR) are also examined through the lens of molecular biology. These conditions not only pose immediate risks to maternal and fetal health, but may also be associated with adverse long-term outcomes. Molecular studies of the placenta, immune signaling, and endothelial dysfunction are deepening our understanding of the origins of these complications. Past research on preeclampsia has advanced our understanding of its pathogenesis; biomarkers such as PlGF, sFlt-1, and PAPP-A play a prominent role, highlighting the importance of larger prospective studies to explore predictive biomarkers and their molecular mechanisms. Biomarker assessment can offer reliable and cost-effective methods for early detection, prognosis, monitoring, and distinguishing between early- and late-onset PE [8,9]. FGR, on the other hand, primarily involves placental insufficiency and has been associated with abnormal remodeling of the utero-placental arteries as well as with abnormal angiogenesis. Its underlying molecular mediators include vascular endothelial growth factor (VEGF), placental growth factor (PlGF), VEGF receptors, and VEGF binding proteins [10].

In addition, this Special Issue includes research in molecular oncology and immunology, particularly in relation to gynecological cancers. Ovarian and endometrial cancers are among the most common gynecological malignancies, often diagnosed at advanced stages. Newer risk stratification models aimed at improving treatment algorithms for patients with endometrial and ovarian cancer are centered around molecular classification. Molecular profiling has revealed critical oncogenic pathways, resistance mechanisms, and immune evasion strategies employed by tumors. These insights are increasingly being applied to design targeted therapies and immunotherapies with the potential to improve outcomes in difficult-to-treat cancers [11,12].

Taken together, the articles in this Special Issue underline the transformative potential of molecular science in understanding, diagnosing, and managing obstetrical and gynecological disorders. While many challenges remain, including standardization of biomarkers, ethical considerations in genetic testing, and ensuring access to molecular diagnostics, the progress presented here is both promising and inspiring.

We hope that this collection stimulates further interdisciplinary research and fosters collaboration between molecular scientists, clinicians, and data analysts. Only through such integrated efforts can we continue to unravel the complexity of women’s health and translate molecular discoveries into clinical benefits.

We would like to express our sincere gratitude to all contributing authors for their high-quality submissions and to the reviewers for their thoughtful evaluations. Special thanks are also due to the editorial team at MDPI for their support in assembling this Special Issue.
